# Ultrafast deep learning super-resolution single-shot T2-weighted imaging for robust edema visualization in cardiovascular magnetic resonance

**DOI:** 10.1016/j.jocmr.2026.102708

**Published:** 2026-03-07

**Authors:** Taraneh Aziz-Safaie, Christoph Katemann, Johannes M. Peeters, Oliver M. Weber, Leon M. Bischoff, Dmitrij Kravchenko, Narine Mesropyan, Thomas M. Vollbrecht, Lucia D. Beissel, Claus C. Pieper, Daniel Kuetting, Julian A. Luetkens, Alexander Isaak

**Affiliations:** a Department of Diagnostic and Interventional Radiology, University Hospital Bonn, Bonn, Germany; b Quantitative Imaging Laboratory Bonn, Bonn, Germany; c Philips GmbH Market DACH, Hamburg, Germany; d Philips MR Clinical Science, Netherlands

**Keywords:** Cardiovascular magnetic resonance, Myocardial edema, T2-STIR magnetic resonance imaging, Cardiac imaging techniques, Deep learning, Image reconstruction

## Abstract

**Background:**

To compare the diagnostic quality of deep learning (DL) super-resolution reconstructed breath-hold (BH) and free-breathing (FB) single-shot (SSH) black-blood T2-weighted short tau inversion recovery (STIR) imaging with standard BH T2-STIR in cardiovascular magnetic resonance (CMR).

**Methods:**

In this prospective study, short-axis BH and FB SSH T2-STIR were added to a standard cardiomyopathy CMR protocol at 1.5T, and DL super-resolution reconstruction was performed. Two readers evaluated diagnostic quality and certainty using a five-point Likert scale. Presence of focal edema was assessed on T2-weighted sequences, including standard T2-STIR and T2 mapping (both used for reference clinical assessment) as well as SSH T2 STIR and DL-SSH T2-STIR. Friedman test and one-way ANOVA were performed.

**Results:**

Eighty-one participants (mean age: 54 ± 20 years; 50 men) were included. No difference was found in edema detection between reference assessment and DL-SSH T2-STIR (both 26%, (21/81 participants)). Scan time was reduced by 63% for BH and 86% for FB DL-SSH T2-STIR compared to standard T2-STIR (90 ± 6 s vs. 35 ± 3 s vs. 243 ± 16 s; p<.0001). BH and FB DL-SSH T2-STIR achieved lower artifact burden (5 [IQR, 4–5] vs. 4 [IQR, 4–5] vs. 4 [IQR, 3–5]; p<.0001), superior image contrast and sharpness compared to standard T2-STIR, especially in non-cooperative or arrhythmic participants. BH and FB DL-SSH T2-STIR imaging provided higher diagnostic certainty than standard T2-STIR (5 [IQR, 5–5] vs. 5 [IQR, 5–5] vs. 4 [IQR, 4–5]; p<.0001). Edema visibility was superior in BH DL-SSH compared to BH-SSH and standard T2-STIR (5 [IQR, 4.8–5] vs. 4 [IQR, 3.3–5] vs. 4 [IQR, 3–4.8]; p<.0001). Inter-rater agreement was substantial to excellent in the rating of edema visibility (BH DL-SSH T2-STIR, κ: 0.73 [95% CI: 0.44–1.0]; BH SSH T2-STIR, κ: 0.79 [95% CI: 0.66–0.97]; standard T2-STIR, κ: 0.86 [95% CI: 0.71–1.0]). Slice level-analysis showed that BH DL-SSH T2-STIR consistently provided superior image quality in apical slices compared to BH SSH and standard T2-STIR (4 [IQR, 4–5] vs. 4 [IQR, 4–4] vs. 4 [IQR, 3–4]; p<.0001).

**Conclusion:**

DL-SSH imaging enabled ultrafast T2-STIR acquisition and robust edema assessment in routine clinical CMR.

## Graphical abstract

**Figure:**
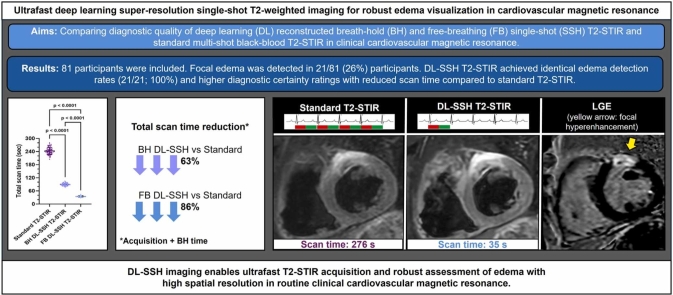

## 1 Introduction

In cardiovascular magnetic resonance (CMR), T2-weighted short tau inversion recovery (T2-STIR) imaging is essential for the assessment of edema in the diagnostic work-up of acute myocardial conditions such as acute myocarditis, ischemic heart disease, pericarditis, takotsubo syndrome, and other non-ischemic cardiomyopathies such as sarcoidosis [1], [2], [3]. Moreover, the presence of edema in cardiomyopathies has prognostic implications [4], [5], [6].

Conventional multi-shot (also known as segmented) T2-STIR imaging can provide high image quality but requires multiple breath-holds and heartbeats to cover the entire heart, which requires patient compliance and significantly impacts patient comfort. Although widely used, multi-shot breath-hold (BH) T2-STIR sequences have certain limitations, such as relatively long acquisition times and susceptibility to heart rate variations and motion [7]. These issues are particularly problematic in patients with limited breath-hold capacity or arrhythmias, where artifacts—such as bright signal from areas of slow flow or signal dropout due to motion—can compromise image quality and potentially mimic or obscure edema.

A variety of CMR techniques are available for the detection of myocardial edema, including segmented T2-weighted short tau inversion recovery, T2-prepared balanced steady-state free precession, and quantitative T2 mapping. Previous studies have demonstrated that single-shot T2-weighted imaging may offer an accelerated alternative in the assessment of myocardial edema [7], [8], [9]. Single-shot techniques can acquire images of the heart within a single heartbeat, enabling quick and efficient assessment of the heart with the option of free-breathing (FB) acquisition. All in all, this fast acquisition, especially in single-shot imaging typically requires reduced acquisition matrix and prolonged echo trains, resulting in compromised image quality, manifested by reduced spatial resolution, blurring, and reduced signal-to-noise ratio.

More recent, different deep learning (DL) approaches have gained increasing attention in CMR, including for T2-weighted imaging. Previous studies have applied DL to denoise T2-STIR sequences for image quality improvement [10] or to accelerate image acquisition by integrating DL modules into compressed sensing [11]. In the context of T2 mapping, DL-based methods have primarily focused on improving segmentation and quantitative accuracy [12], [13].

While these studies highlight the utility of different DL approaches in T2-weighted CMR, they typically operate within the spatial resolution limits of the acquired data and do not incorporate super-resolution strategies, which could be particularly beneficial in single-shot T2-STIR imaging, which is sensitive to through-plane artifacts and partial volume effects—especially in apical slices and thin-walled myocardium. Thus, DL-based super-resolution reconstruction of single-shot T2-STIR imaging in a clinical patient cohort remains largely unexplored. The present study investigates such an approach, evaluating its impact on edema visibility and diagnostic performance in comparison with both standard segmented and non–DL-reconstructed single-shot T2-STIR sequences with the option of free-breathing acquisition.

## 2 Materials and methods

The institutional ethics committee approved this prospective study, and all participants gave informed consent (study ID: 2024–379-BO). From February 2024 to May 2024, participants with a clinical indication for CMR were included in the study and underwent additional single-shot T2-STIR sequences with free-breathing and breath-holding. Exclusion criteria applied before recruitment included general contraindications to MRI, severe claustrophobia, or being underage. Only participants with both standard and single-shot T2-STIR were included for final analysis. The demographics are provided in Table 1.

**Table 1:** General characteristics and cardiovascular magnetic resonance (CMR)-based diagnosis of included participants (n = 81)

General characteristics	
Age (years)	54± 20
Sex (males)	50 (61%)
Weight (kg)	79± 18
Height (cm)	174± 11
Body mass index (kg/m^2^)	26 ± 5
Body surface area (m^2^)	2± 0.3
Heart rate (beats/min)	70± 15
*CMR diagnosis*	
Presence of myocardial or pericardial edema	21 (26%)
Ischemic cardiomyopathy	11 (14%)
(Sub-)acute infarction	3 (27%)
Old ischemic scare	8 (73%)
Non-ischemic cardiomyopathy	37 (46%)
Dilated cardiomyopathy	6 (16%)
Hypertrophic cardiomyopathy	11 (30%)
Acute myocarditis or pericarditis	9 (24%)
Post myocarditis/ non-ischemic scar	5 (14%)
Amyloidosis	4 (11%)
Peripartum cardiomyopathy	1 (3%)
Cardiac manifestation of systemic lupus erythematosus	1 (3%)
Segmental, slight left-ventricular hypertrophy	3 (4%)
Valvular disease	4 (5%)
Dyssynchronous contractility due to arrhythmia	2 (3%)
Other unspecific findings	10 (12%)
No pathological findings	14 (17%)

Data are numbers (%) of cases or means ± standard).

### 2.1 Image acquisition

**Fig. 1:**
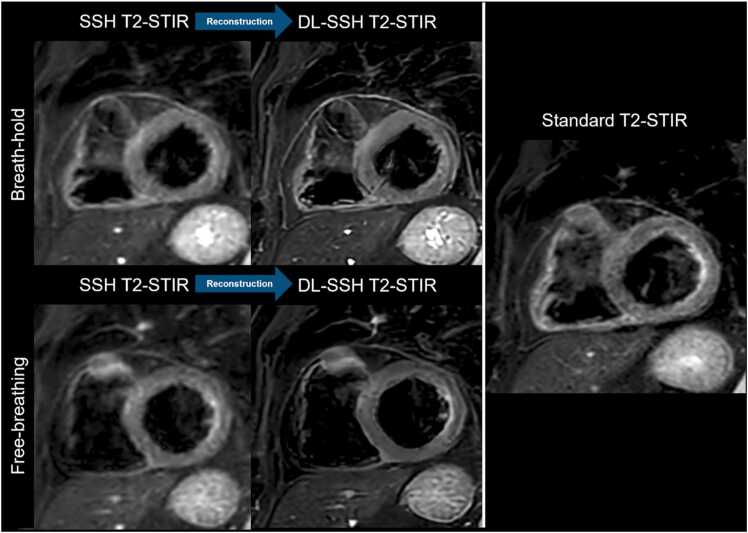
Cardiovascular magnetic resonance imaging examples of a 62-year-old participant with follow-up of pericarditis showing standard breath-hold multi-shot T2-weighted STIR sequence (standard T2-STIR), conventional SSH T2-STIR, and DL-SSH T2-STIR acquired with breath-hold and free-breathing. *STIR* short tau inversion recovery, *SSH T2-STIR* single-shot T2-STIR, *DL-SSH T2-STIR* deep learning reconstructed single-shot T2-STIR

Examinations were performed on a clinical whole-body MRI system (Ingenia 1.5 Tesla; Philips Healthcare, Best, the Netherlands) using a 16-channel body coil and a 12-channel table coil with digital interface for signal reception. The CMR examination protocol incorporated standard sequences for the evaluation of non-ischemic or ischemic cardiomyopathies: cine imaging, late gadolinium enhancement (LGE), T1 and T2 mapping (apical, mid-ventricular, basal sections), and T2-weighted sequences. For the latter, two different T2-STIR strategies were used as follows: (1) standard segmented black-blood turbo spin echo breath-hold sequence (standard T2-STIR), acquired with parallel imaging (SENSE); (2) single-shot black-blood T2-STIR sequence (SSH T2-STIR), accelerated with Compressed sensitivity encoding (CS-SENSE, a vendor-integrated technique combining SENSE and compressed sensing); with BH and FB. All T2-STIR sequences were acquired in short-axis orientation from base to apex. To ensure identical anatomical coverage, the slice planning was performed once and copied across all T2-STIR sequences without adjustment. For detailed parameters, see Table 2 for representative imaging examples of the method, see Fig. 1. Additional sequences were used tailored to each patient’s clinical indication. The T2-STIR sequences are used in our routine clinical practice (standard T2-STIR for stable patients and SSH T2-STIR for challenging examinations of patients with insufficient breath-hold capacities and/ or arrhythmia) and enable a controlled comparison of acceleration strategies as follows: SENSE in standard T2-STIR imaging, Compressed SENSE in single-shot T2-imaging, and Compressed SENSE combined with DL super-resolution in DL-SSH T2-STIR. DL image reconstruction was performed on the clinical MRI scanner during the CMR scan (directly after acquiring the SSH T2-STIR) using a vendor-provided prototype software algorithm. The super-resolution DL framework integrates compressed sensing with two convolutional neural networks: Adaptive-CS-Net [14] and Precise-Image-Net [15]. Adaptive-CS-Net removes noise and undersampling artifacts during coil combination while ensuring data consistency through multiscale sparsification. Precise-Image-Net enhances matrix size and image sharpness while eliminating ringing artifacts and replacing zero-filling. The super-resolution DL network was trained on six million image pairs of high-resolution and downscaled images to optimize ringing artifact removal and resolution enhancement, as previously described [16], [17]. In our study and in the literature, “super-resolution” specifically refers to reconstructing higher-resolution images from relatively low-spatial-resolution single-shot data, not to increasing the true acquired resolution [15], [16]. Only the non-industry authors had full control over the data in this study.

**Table 2:** Imaging parameters of standard T2-STIR black-blood turbo spin echo breath-hold sequence and of the single-shot black-blood T2-STIR sequence with both breath-hold and free-breathing

	Standard T2-STIR	BH Single-Shot T2-STIR	FB Single-Shot T2-STIR
Field of view (mm^2^)	250 × 250	250 × 250	250 × 250
Repetition time (ms)	1176	3000	3000
Echo time (ms)	70	64	64
Flip angle (degree)	90	90	90
In-plane resolution, acquired (mm^2^)	1.60 × 2.07	2.02 × 2.52	2.02 × 2.52
In-plane resolution, reconstructed (mm^2^)	0.87 × 0.87	0.87 × 0.87	0.87 × 0.87
Slice thickness (mm)	8	8	8
TSE echo spacing/shot (ms)	5.6/134	3.4/124	4.2/156
TSE factor	24	37	37
Inversion Delay (ms)	165	165	165
SENSE/Compressed SENSE Factor	2.5/ -	- /4	- /4

Deep learning image reconstruction was performed after acquiring single-shot cine on the clinical MRI scanner. *BH* Breath-hold, *FB* free-breathing, *TSE* turbo spin echo, *SENSE* sensitivity encoding, *STIR* short tau inversion recovery

### 2.2 Image quality analysis

Two cardiovascular radiologists (T.A.S. and A.I.; 3 and 7 years of experience in CMR) independently evaluated subjective image quality as well as the presence of focal myocardial or pericardial edema in all three sequences. Sequences were presented in random order.

### 2.3 Objective image quality parameters

Apparent signal-to-noise ratio (aSNR) was determined by dividing the mean signal intensity of the myocardium by the standard deviation of the signal intensity in skeletal muscle. Apparent contrast-to-noise ratio (aCNR) was obtained in cases with focal myocardial or pericardial edema by subtracting the mean edema signal intensity from the mean myocardial signal intensity, then dividing that difference by the standard deviation of the skeletal muscle signal intensity. Furthermore, the quality of black-blood preparation was compared by subtracting the mean myocardial signal intensity from the mean blood-pool signal intensity, then dividing that difference by the standard deviation of the skeletal muscle signal intensity. The T2 signal intensity ratio was calculated by dividing the mean myocardial signal intensity by the mean skeletal muscle signal intensity. Furthermore, edge rise distance was measured in 50 participants as an indicator of image sharpness, following previously described methods [16], [18]. This measurement was performed using custom in-house software developed in MATLAB (Version R2022b, MathWorks, Natick, Massachusetts).

### 2.4 Subjective image quality parameters

Subjective image quality was assessed for both the standard T2-STIR and DL-SSH T2-STIR sequences using a five-point Likert scale based on three criteria as follows: blood-pool–myocardium contrast, endocardial edge definition, and artifact burden. For blood pool to myocardium contrast, the ratings were: 1 = non-diagnostic, 2 = poor contrast, 3 = intermediate contrast, 4 = good contrast, and 5 = excellent contrast. Endocardial edge definition was evaluated as follows: 1 = non-diagnostic, 2 = washed-out endocardial edge, 3 = intermediate delineation, 4 = good definition with minimal blurring, and 5 = clear delineation. Artifact burden was rated as: 1 = non-diagnostic, 2 = numerous artifacts, 3 = moderate artifacts, 4 = minimal artifacts, and 5 = no artifacts. Additionally, overall image quality was independently assessed for basal, mid-ventricular, and apical slices using a 5-point Likert scale (1 = non-diagnostic, 2 = poor quality, 3 = intermediate quality, 4 = good quality, and 5 = excellent quality) for standard T2-STIR, BH SSH, and BH DL-SSH T2-STIR to evaluate potential differences in performance across the heart slices. The total score for each of the four criteria was calculated as the equally weighted average of the ratings from the two raters.

### 2.5 Diagnostic evaluation of edema

An experienced cardiovascular radiologist (JAL; 12 years of experience in CMR), blinded to the DL-SSH T2-STIR sequences, reviewed and interpreted the CMR examinations within the routine clinical workflow, including the assessment of focal myocardial and pericardial edema using standard multi-shot breath-hold T2-STIR imaging and T2 mapping (reference standard). Final CMR-based diagnoses, including the assessment of focal myocardial and pericardial edema are listed in Table 1. Two cardiovascular radiologists (T.A.S. and A.I.) reviewed the presence of focal edema in consensus on three imaging sets, including cine and late gadolinium enhancement images with (I) standard T2-STIR, (II) BH DL-SSH T2-STIR, and (III) FB DL-SSH T2-STIR (without the review of T2 mapping; washout period eight weeks, respectively). Additionally, subjective diagnostic confidence was rated for each sequence by the two raters using a five-point Likert scale (1 = non-diagnostic, 2 = poor confidence, 3 = intermedium confidence, 4 = good confidence, 5 = high confidence). In cases with presence of edema both readers independently evaluated the visibility of focal edema on a five-point Likert scale for standard T2-STIR, BH SSH T2-STIR, and BH DL-SSH T2-STIR (1 = minimal visibility, 2 = low visibility, 3 = moderate visibility, 4 = good visibility, and 5 = excellent visibility).

### 2.6 Statistical analysis

Statistical analysis was carried out using Prism (version 9.5.1; GraphPad Software, San Diego, California) and SPSS (version 29; IBM, Armonk, New York). Data distribution was evaluated with histograms and the Shapiro–Wilk test. Continuous variables for quantitative measurements are presented as mean ± standard deviation, while nominal data are shown as percentages with absolute frequencies. For nonparametric data or when normality could not be confirmed, medians and interquartile ranges (IQR) are reported. Differences in subjective image scores, edema visibility, diagnostic certainty, and objective image scores among the three sequences were analyzed using the Friedman test, whereas one-way analysis of variance (ANOVA) was applied for acquisition time and T2 signal-intensity ratio. Post hoc comparisons were conducted using Dunn’s and Holm-Šídák tests to identify specific group differences. Inter-rater agreement for overall subjective image quality—which encompassed combined contrast, artifacts, and endocardial edge definition as well as edema visibility was assessed using Cohen’s weighted κ (<0.21 = poor, 0.21–0.40 = fair, 0.41–0.60 = moderate, 0.61–0.80 = substantial, and 0.81–1.0 = excellent). Diagnostic accuracy of myocardial edema detection in DL-SSH T2-STIR was assessed using contingency table analysis calculating sensitivity and specificity with T2 mapping as the reference standard for focal myocardial edema detection. Statistical significance was defined as p<.05.

## 3 Results

### 3.1 Participants and CMR characteristics

Eighty-one participants (mean age: 54 ± 20 years; 50 men) were included in this study; participant characteristics as well as the CMR diagnosis are summarized in Table 1. Clinical indications for CMR included suspicion or follow-up of inflammatory cardiac disease like myocarditis or pericarditis (40.7%, 33/81), suspicion of dilated or hypertrophic cardiomyopathy 14.8%, 12/81), reduced ejection fraction of unknown cause 11.1%, 9/81), suspicion or history of sarcoidosis/amyloidosis 8.6%, 7/81), and evaluation of ischemic cardiomyopathy 6.2%, 5/81). Other indications 7.4%, 6/81) included evaluation of structural heart disease as a cause of arrhythmia, recurrent syncope, mitral annulus disjunction, pre-therapy assessment, and pericardial effusion of unknown cause.

**Fig. 2:**
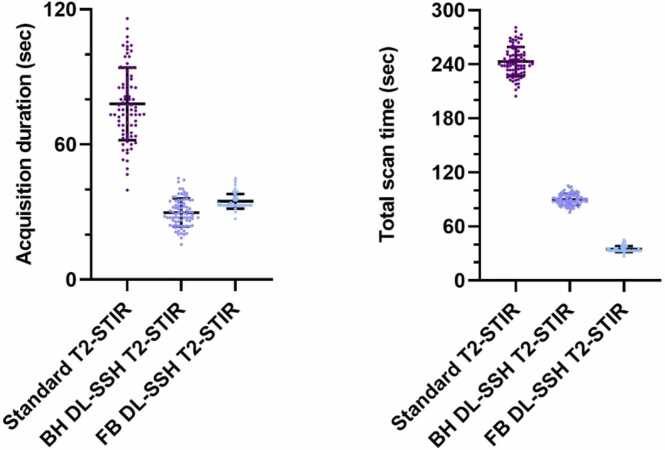
Scatter plot showing mean and standard deviation, depicting significant accelerated acquisition duration (without breath-hold time) and total scan time (including breath-hold time) for DL-SSH T2-STIR with BH and FB compared to the standard T2-STIR black-blood (standard T2-STIR) sequence (all p<.0001). *DL-SSH T2-STIR* deep learning reconstructed single-shot T2-STIR black-blood imaging, *BH* breath-hold, *FB* free-breathing

The mean total scan time (including both acquisition duration and breath-hold time) of BH DL-SSH T2-STIR was 63% lower and that of FB DL-SSH T2-STIR was 86% lower compared to standard T2-STIR (90 ± 6 s vs. 35 ± 3 vs. 243 ± 16 s; p<.0001; see Fig. 2). The mean acquisition duration of BH DL-SSH T2-STIR (time without breath-holding) was 62% lower and that of FB DL-SSH T2-STIR and was 55% lower compared to standard T2-STIR (29 ± 6 s vs. 35 ± 3 vs. 78 ± 16 s; p<.0001; see Fig. 2). For the full short-axis stack, the standard T2-STIR sequence was acquired with 12 breath-holds (1 slice per breath-hold), whereas BH SSH T2-STIR sequence was acquired with 4 breath-holds (3 slices per breath-hold). The average duration for the DL reconstruction of a full stack (12 slices) of SSH T2-STIR images on the clinical MRI system was 10 ± 1 s.

### 3.2 Qualitative and quantitative image analysis results

**Fig. 3:**
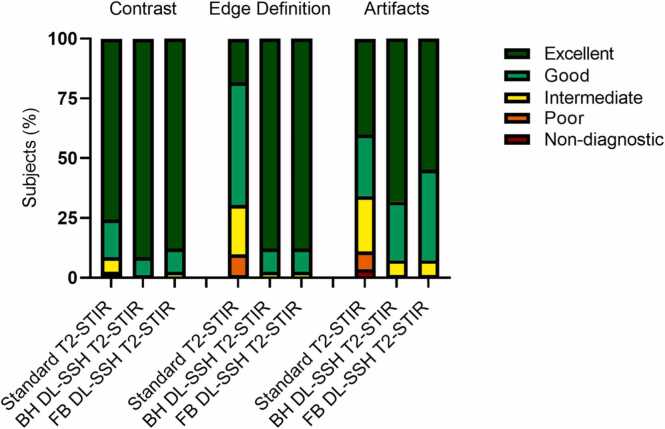
Stacked bar charts show the distribution of Likert scale scores used for qualitative image assessment of DL-SSH T2-STIR with BH and FB compared to the standard T2-STIR black-blood (standard T2-STIR) sequence. *Edge definition* Endocardial edge definition, *DL-SSH T2-STIR* deep learning reconstructed single-shot T2-STIR black-blood imaging, *BH* breath-hold, *FB* free-breathing

**Fig. 4:**
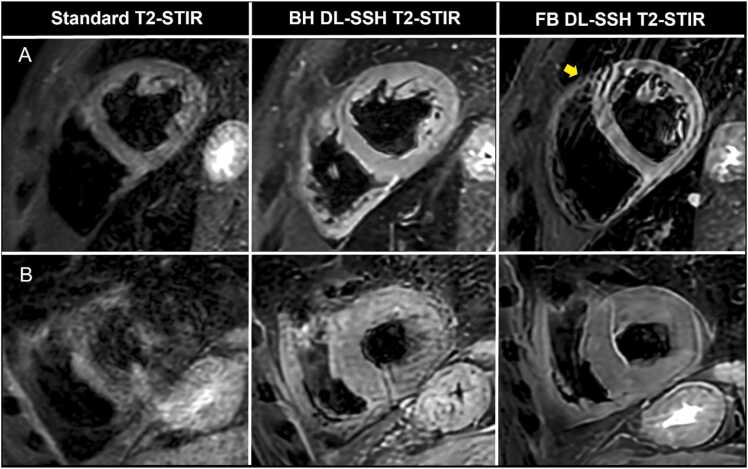
Cardiovascular magnetic resonance imaging examples depicting artifacts in DL-SSH T2-STIR acquired with BH and FB and standard T2-STIR. (A) 66-year-old participant with sufficient breath-holding capacity; vertically oriented linear artifacts in FB DL-SSH T2-STIR, which most likely represent ghost artifacts of the chest wall due to excessive chest wall movement (yellow arrow). (B) 35-year-old participant with inconsistent breath-holding and distinctly better diagnostic quality of FB DL-SSH T2-STIR compared to both breath-holding sequences (non-diagnostic quality of standard T2-STIR). *DL-SSH T2-STIR* deep learning reconstructed single-shot T2-STIR black-blood imaging, *BH* breath-hold, *FB* free-breathing

Summarized results for the subjective and objective image quality assessment are listed in Table 3. The distribution of subjective image quality scores is shown in Fig. 3. The comparison of contrast ratings showed significantly higher contrast ratings for BH DL-SSH and FB DL-SSH T2 compared to standard T2-STIR (5 [IQR, 5–5] vs. 5 [IQR, 5–5] vs. 5 [IQR, 4–5]; p=.0002); see Table 3 for group-wise comparison. There were no significant differences in the comparison of aCNR, aSNR, quality of black-blood preparation, and T2 signal-intensity ratio between the three sequences (Table 3). Artifact analysis demonstrated better ratings for BH DL-SSH compared to FB DL-SSH and standard T2-STIR (5 [IQR 4–5] vs. 4 [IQR 4–5] vs. 4 [IQR 3–5]; p<.0001); see Table 3. Although no significant difference was found in the artifact burden between BH and FB DL-SSH T2-STIR, there were some differences in the type of observed artifacts. In individual participants with inconsistent breath-holding, fewer artifacts were observed in FB DL-SSH T2-STIR compared to both standard T2-STIR and BH DL-SSH T2-STIR. In some participants without breath-holding issues motion artifacts were observed on FB compared to BH DL-SSH T2-STIR; see Fig. 4 for representative image examples.

**Table 3:** Results from comparison of objective and subjective image quality in deep learning reconstructed single-shot T2-STIR black-blood imaging with breath-hold (BH) and free-breathing (FB) and standard T2-STIR black-blood imaging

Variable	Standard T2-STIR	BH DL-SSH T2-STIR	FB DL-SSH T2-STIR	p value, between all groups	p value, BH vs SD	p value, FB vs SD	p value, BH vs FB
*Objective Metrics*							
aSNR	22.4 [16.7–28.6]	23.9 [17.6–33.9]	25.3 [17.3–30.9]	.08	-	-	-
aCNR	21.6 [6.5–26.8]	20.6 [7.4–27.5]	19.5 [8.0–26.7]	.82	-	-	-
BB preparation quality	19.6 [14.2–24.4]	21.7 [14.8–27.7]	21.1 [15.5–30.1]	.11			
T2 SI ratio	1.8±0.4	1.9±0.4	1.9±0.5	.09	-	-	-
Edge rise distance (mm)	2.4 [2.1–3.2]	1.4 [1.2–1.7]	1.5 [1.3–1.8]	<.0001	<.0001	<.0001	>.99
*Subjective Metrics*							
Contrast	5[4–5]	5[5]	5[5]	.0002	.07	.41	>.99
Endocardial edge definition	4 [3.3–4]	5[5]	5[5]	<.0001	<.0001	<.0001	>.99
Artifacts	4[3–5]	5[4–5]	4[4–5]	<.0001	<.0001	.06	.06
Diagnostic certainty	4[4–5]	5[5]	5[5]	<.0001	<.0001	<.0001	>.99

Friedman test and post hoc pairwise comparisons using Dunn’s multiple comparison test were used to identify specific group differences unless otherwise noted. One-way ANOVA was applied for T2 signal-intensity ratio. Median and interquartile range are provided for aSNR, aCNR, BB preparation quality, and subjective metrics. Mean and standard deviation are provided for T2 signal-intensity ratio. *aSNR* apparent signal to noise ratio, *aCNR* apparent contrast to noise ratio, *BB* Black blood, *T2 SI* T2 signal-intensity ratio, *STIR* Short tau inversion recovery, *DL* Deep learning, *SSH* Single-shot, *BH* Breath-hold, *FB* Free-breathing, *SD* Standard T2-STIR. Data are means ± standard deviation or medians (interquartile range).

In the analysis of image sharpness, both BH and FB DL-SSH T2-STIR achieved significantly better results than standard T2-STIR for endocardial edge definition ratings (5 [IQR, 5–5] vs. 5 [IQR, 5–5] vs. 4 [IQR, 3–4]; p<.0001) and edge rise distance (1.4 [IQR, 1.2–1.7] vs. 1.5 [IQR, 1.3–1.8] vs. 2.4 [IQR, 2.1–3.2] mm; p<.0001); see Table 3.

**Fig. 5:**
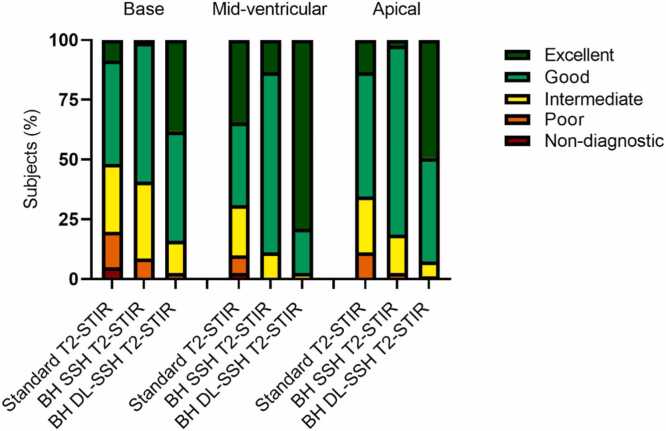
Stacked bar charts show the distribution of Likert scale scores used for overall qualitative image assessment of DL-SSH T2-STIR with BH compared to the standard T2-STIR black-blood (standard T2-STIR) sequence and single-shot T2-STIR black-blood imaging (SSH T2-STIR) with breath-hold of basal, mid-ventricular, and apical slices. *DL-SSH T2-STIR* deep learning reconstructed single-shot T2-STIR black-blood imaging, *BH* breath-hold

Slice-level analysis indicated that image quality at the apical level was superior in BH DL-SSH T2-STIR compared to BH SSH and standard T2-STIR (4 [IQR, 4–5] vs. 4 [IQR, 4–4] vs. 4 [IQR, 3–4]; p<.0001). Image quality in BH DL-SSH T2-STIR at mid-ventricular and basal level was also superior compared to standard T2-STIR imaging; see Table 4 and Fig. 5 and S1).

**Table 4:** Results from comparison of overall image quality of basal, mid-ventricular and apical slices in deep learning reconstructed single-shot T2-STIR black-blood imaging with breath-hold (BH), single-shot T2-STIR imaging with breath-hold and standard T2-STIR black-blood imaging

Variable	Standard T2-STIR	BH SSH T2-STIR	BH DL-SSH T2-STIR	p value, between all groups	p value, DL vs SD	p value, DL vs SSH	p value, SD vs SSH
Basal	4[3–4]	4[3–4]	4[4–5]	<.0001	<.0001	<.0001	>.99
Mid-ventricular	4[3–5]	4[4]	5[5]	<.0001	<.0001	<.0001	>.99
Apical	4[3–4]	4[4]	4[4–5]	<.0001	<.0001	<.0001	>.99

Friedman test and post hoc pairwise comparisons using Dunn’s multiple comparison test were used to identify specific group differences unless otherwise noted. Median and interquartile range are provided for STIR. *STIR* Short tau inversion recovery, *DL* Deep learning, *SSH* Single-shot, *BH* Breath-hold. Data are medians (interquartile range).

Inter-rater agreement was excellent in the rating of overall subjective image quality including combined contrast, artifact und endocardial edge definition scores for standard T2-STIR (κ: 0.95 [95% CI: 0.91–0.98]), BH DL-SSH T2-STIR (κ: 0.93 [95% CI: 0.87–0.99]) and FB DL-SSH T2-STIR (κ: 0.92 [95% CI: 0.86–0.98]) imaging.

### 3.3 CMR assessment of myocardial and pericardial edema

**Fig. 6:**
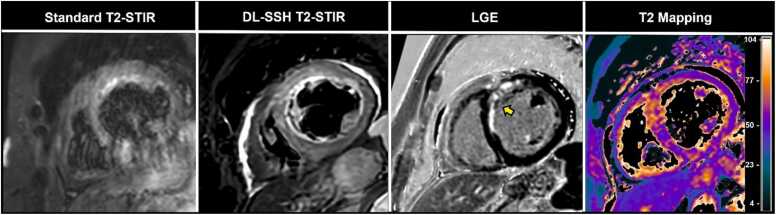
Cardiovascular magnetic resonance imaging examples in short-axis view from a 62-year-old participant after interventional treatment for occlusion of the left anterior descending coronary artery show subacute myocardial infarction. Myocardial edema is not clearly assessable in the standard T2-STIR images due to severe motion artifacts, due to the limited compliance of the patient. DL-SSH T2-STIR and T2-mapping clearly depict focal edema of the anterior and anterior-septal wall with corresponding LGE (yellow arrow), indicating subacute infarction. *Standard T2-STIR* Breath-hold multi-shot T2-weighted short tau inversion recovery sequence; *DL-SSH T2-STIR* deep learning reconstructed single-shot T2-STIR; *LGE* late gadolinium enhancement

**Fig. 7:**
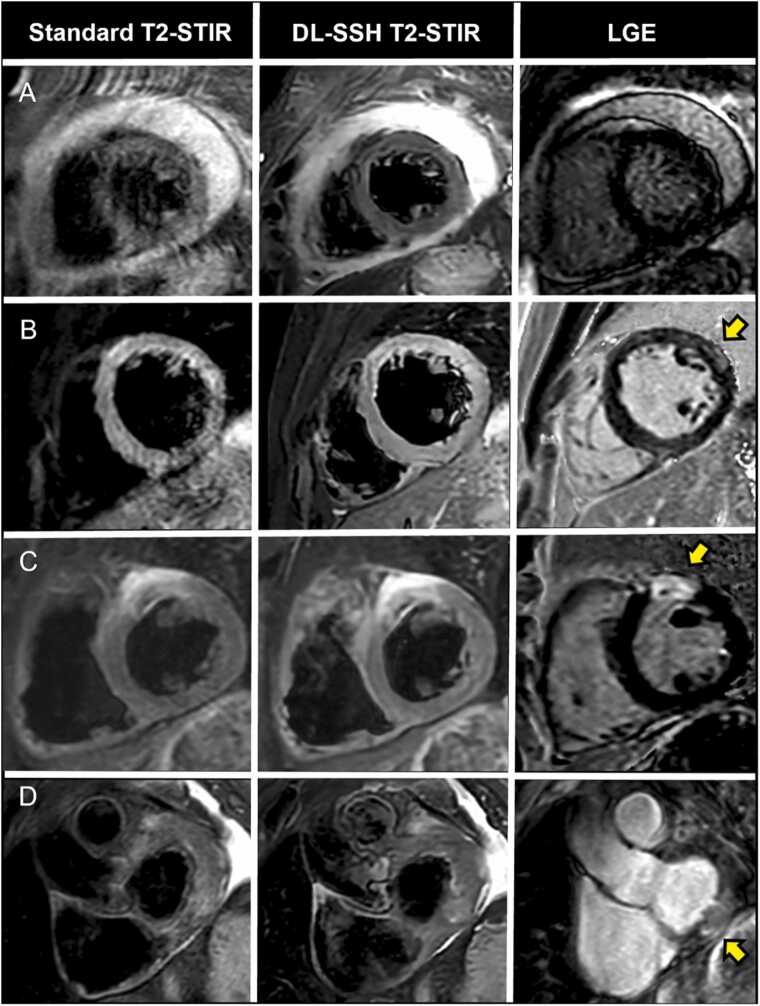
Cardiovascular magnetic resonance imaging examples in (A) a 62-year-old participant with purulent pericarditis, in (B) a 17-year participant with acute myocarditis, in (C) a 57-year participant with (sub) acute myocardial infarction and in (D) a 73-year participant with myocardial metastasis from renal cell carcinoma. Yellow arrows: Focal hyperenhancement. *Standard T2-STIR* Breath-hold multi-shot T2-weighted short tau inversion recovery sequence, *DL-SSH T2-STIR* deep learning reconstructed single-shot T2-STIR, *LGE* late gadolinium enhancement

On reference assessment (without review of DL-SSH T2-STIR), focal edema was found in 26% (21/ 81) participants, of whom 57% (12/21) had myocardial edema and 43% (9/21) (43%) had pericardial edema (see Table 1). In the study-related diagnostic assessment of the three sequences, edema was detected in 95% (20/21) participants with edema in the reference assessment on standard T2-STIR and in 100% (21/21) participants on both BH and FB DL-SSH T2-STIR. In the case where focal myocardial edema was not detected on standard T2-STIR and in SSH T2-STIR but was visible on both BH and FB DL-SSH T2-STIR, the participant had an arrhythmia with impaired image quality on standard imaging. In the reference diagnostic reading, T2 mapping confirmed the presence of focal myocardial edema (see also Fig. 6 for representative images). There was complete agreement between the reference assessment and the DL-SSH T2-STIR assessment of focal edema (21/21 participants [100%]). Furthermore, subjective edema visibility was rated significantly higher in BH DL-SSH T2-STIR compared to BH SSH and standard T2-STIR (5 [IQR, 4.8–5] vs. 4 [IQR, 3.3–5] vs. 4 [IQR, 3–4.8; p<.0001). BH and FB DL-SSH T2-STIR sequences received higher diagnostic confidence ratings for edema assessment than standard T2-STIR (5 [IQR, 5–5] vs. 5 [IQR, 5–5] vs. 4 [IQR, 4–5]; p<.0001). Exemplary clinical images of participants with edema are illustrated in Fig. 7.

Inter-rater agreement was excellent in the rating of edema visibility in standard T2-STIR (κ: 0.86 [95% CI: 0.71–1.0]) and substantial in BH SSH T2-STIR (κ: 0.79 [95% CI: 0.66–0.97) and BH DL-SSH T2-STIR (κ: 0.73 [95% CI: 0.44–1.0]) imaging.

Among the 12 participants with focal myocardial edema detected on DL-SSH T2-STIR, 92% (11/12) demonstrated elevated focal T2 mapping values. In one case with focal edema on DL-SSH T2-STIR at the very basal–inferolateral segment, T2 mapping values were borderline-normal, but the focal region was not included in the short-axis coverage of the mapping slices. Of the 9 participants with pericardial edema, 22% (2/9) showed elevated global T2 mapping values consistent with diffuse myocardial edema, which might be related to diffuse myocardial involvement in pericarditis. Among the 60 participants without visually detected edema on T2-STIR, no focal edema was detected on T2 mapping. However, 15% (9/60) exhibited elevated global myocardial T2 relaxation times, corresponding to pathologies with diffuse myocardial involvement such as cardiac amyloidosis (n = 3), ischemic cardiomyopathy (n = 1), or suspected diffuse myocardial inflammation (n = 5). In a contingency table analysis comparing focal myocardial edema detection between DL-SSH T2-STIR and focal T2 mapping values, DL-SSH T2-STIR demonstrated a sensitivity of 100% and a specificity of 98.4% for focal myocardial edema detection.

## 4 Discussion

In this study, we applied a deep learning super-resolution algorithm to reconstruct single-shot T2-STIR images acquired with both breath-holding and free-breathing and compared their diagnostic quality to standard segmented, breath-hold T2-STIR imaging. The acceleration strategies used reflect routine clinical protocols: standard segmented T2-STIR is typically employed in stable patients, while single-shot T2-STIR can be used in patients with limited breath-hold capacity or arrhythmia. Deep learning reconstruction was applied to the single-shot acquisitions using a framework based on Adaptive-CS-Net and Precise-Image-Net, enabling both image denoising and true image-space super-resolution after reconstruction. DL-SSH T2-STIR showed overall non-inferior diagnostic quality with even superior image contrast and sharpness as well as lower artifact burden, while cutting total scan time by 63% for breath-holding DL-SSH T2-STIR and by 86% for free-breathing DL-SSH T2-STIR compared to standard T2-STIR. No significant differences were observed in the assessment of objective image parameters between all three sequences. Our results show that the main limitation of lower resolution in single-shot T2-STIR imaging can be effectively mitigated using a DL super-resolution algorithm and thereby effectively accelerate clinical cardiomyopathy protocols.

The widely used multi-shot breath-hold T2-STIR has some limitations, such as relatively long acquisition times and susceptibility to heart rate variations and to motion artifacts [7]. Thus, diagnostic quality may suffer in patients with limited breath-hold capacities or arrhythmias, owing to breathing artifacts and myocardial signal loss from motion-induced misalignment of the selective black-blood re-inversion pulse and excitation pulse slice profile. To overcome these limitations, a previous study has employed free-breathing single-shot T2-prepared steady-state free precision sequence for the assessment of acute and chronic myocardial infarction [9]. Here, the authors demonstrated lower artifact burden and better diagnostic quality compared to standard dark-blood T2 turbo spin echo sequence, especially in patients with arrhythmia and with higher heart rates [9]. This is in line with studies that investigated single-shot imaging in other CMR sequences and demonstrated its lower susceptibility to motion-related artifacts [19] as limitation of multi-shot T2-STIR can be mitigated in SSH T2-STIR imaging, where sensitivity to cardiac cycle variations is reduced and may provide more consistent tissue contrast. However, the single-shot T2-prepared steady-state free precision approach showed significant limitations in spatial resolution and objective image parameters of aSNR and aCNR compared to standard T2 black-blood turbo spin echo sequence [9].

Moreover, recent studies have applied deep learning to T2-weighted CMR for artifact reduction and accelerated image acquisition. For instance, Ogawa et al. showed improved border sharpness and SNR using DL-based denoising in segmented T2-black-blood images [10]; but did not test the impact of DL reconstruction on acquisition time. Yan et al. proposed a DL-regularized compressed sensing framework integrated into the k-space reconstruction of breath-hold multi- and single-shot acquisitions. They tested this approach in a clinical cohort of 33 patients, achieving reduced acquisition times by about 80% with a DL single-beat approach, while maintaining comparable diagnostic quality and effectively reducing the number of required breath holds [11].

While our findings are consistent with those of previous studies, our approach extends the applicability of deep learning reconstruction in CMR T2-weighted imaging by incorporating super-resolution and also validating fast free-breathing acquisition. In contrast to prior methods, which primarily focused on denoising or artifact suppression within the limits of the acquired matrix in breath-hold imaging, this technique enhances spatial resolution by reconstructing beyond the native sampling grid.

Based on prior work demonstrating the robustness of our DL super-resolution framework in single-beat cine CMR—where other DL methods have shown reduced edge sharpness or variability in functional analysis of cine imaging within a single-heartbeat [20]—we applied the same framework to single-shot T2-STIR imaging to address its well-recognized spatial resolution limitations [21]. We found that DL-based super-resolution reconstruction can effectively mitigate the spatial limitations of single-shot T2-STIR imaging, offering higher diagnostic image quality and superior edema visibility—also in apical slices or thin-walled myocardium - compared to conventional acceleration techniques such as parallel imaging and compressed sensing. Free-breathing and breath-hold DL-SSH T2-STIR provided reliable visual assessment of myocardial and pericardial edema with even higher diagnostic certainty compared to standard T2-STIR imaging, achieving full diagnostic agreement compared to the reference diagnostic assessment, in a broad spectrum of different cardiac pathologies with focal edema, such as myocarditis, pericarditis, sarcoidosis, myocardial infarction, and hypertrophic-obstructive cardiomyopathy.

As the relatively large number of patients with atrial fibrillation poses a challenge for CMR in clinical practice, this single-shot approach with the possible addition of the free-breathing technique is particularly advantageous [22]. Whereas no significant difference in artifact burden was observed between FB DL-SSH T2-STIR compared to both BH sequences in the total cohort, FB DL-SSH T2-STIR showed better image sharpness and lower artifact burden in some participants with inconsistent breath-holding capacity, while also enabling particularly rapid acquisition times. These features show that the sequence is particularly suitable for patients with challenging conditions. However, it should be noted that in some patients without breath-holding limitations, FB DL-SSH T2-STIR exhibited some motion artifacts compared to the BH DL-SSH T2-STIR, possibly due to excessive thoracic movement. Thus, the choice between a free-breathing or breath-hold approach in DL-SSH T2-STIR should be primarily tailored to the individual patient's needs and ability to ensure optimal image quality.

Another clinical benefit of DL-SSH T2-STIR in the diagnosis of edema is its ability to enhance image sharpness. For myocardial edema, DL-SSH T2-STIR may provide clearer delineation of the myocardium, improving the visualization of edematous tissue to normal myocardium. Improved image sharpness was particularly beneficial for assessing the relatively thin pericardium, facilitating clearer differentiation from neighboring structures, such as the lungs or epicardial fat, and clearer detection of inflammatory changes, thereby contributing to a greater diagnostic certainty in the evaluation of pericarditis.

## 5 Limitations

Our study has some limitations. We focused on the evaluation of multi-slice T2-STIR in the short-axis view only, which is the standard sequence for qualitative edema assessment in most centers. Although long and short axis T2-STIR imaging rely mainly on the same imagining parameters, the impact and benefits of DL-SSH for long axis T2-STIR are beyond the scope of this study. Furthermore, the moderate sample size of participants with myocardial and pericardial edema may limit the generalizability of our findings on the broad spectrum of cardiovascular diseases. The study was conducted at a single center and employed a deep learning-based imaging reconstruction software that is currently in development for clinical implementation. The DL reconstruction used in this study is a vendor-integrated implementation of Adaptive-CS-Net and Precise-Image-Net, directly integrated inline on the MRI system. As this framework is proprietary, the model code and weights cannot be publicly released. However, all acquisition parameters and architectural components have been detailed to support reproducibility, and the reconstruction pipeline is under evaluation for future clinical deployment, enabling external validation once available.

## 6 Conclusion

In conclusion, this study demonstrates that applying DL reconstruction can overcome the common limitations of single-shot T2-STIR imaging. DL-SSH T2-STIR enables ultrafast, reliable, and robust imaging of edema in various myocardial and pericardial diseases, contributing to a fast and robust CMR protocol with the option for free-breathing acquisitions. This approach can enhance patient comfort, improve compliance and image quality, and help meet the growing demand for CMR.

## Author contributions

**Taraneh Aziz-Safaie:** Writing – review & editing, Writing – original draft, Visualization, Investigation, Formal analysis. **Christoph Katemann:** Writing – review & editing, Methodology. **Johannes M. Peeters:** Writing – review & editing. **Oliver M. Weber:** Writing – review & editing. **Leon M. Bischoff:** Writing – review & editing. **Dmitrij Kravchenko:** Writing – review & editing. **Narine Mesropyan:** Writing – review & editing. **Thomas M. Vollbrecht:** Writing – review & editing. **Lucia D. Beissel:** Writing – review & editing. **Claus C. Pieper:** Writing – review & editing. **Daniel Kuetting:** Writing – review & editing. **Julian A. Luetkens:** Writing – review & editing, Supervision. **Alexander Isaak:** Writing – review & editing, Visualization, Supervision, Investigation, Conceptualization.

## Declaration of competing interests

The authors declare the following financial interests/personal relationships which may be considered as potential competing interests: Christoph Katemann reports a relationship with Philips GmbH that includes employment. Oliver M. Weber reports a relationship with Philips GmbH that includes employment. Johannes M. Peeters reports a relationship with Philips GmbH that includes employment. Dmitrij Kravchenko reports a relationship with Philips GmbH that includes speaking and lecture fees. Dmitrij Kravchenko reports a relationship with Elucid Bioimaging that includes consulting or advisory. Alexander Isaak reports a relationship with Philips GmbH that includes speaking and lecture fees. Julian A. Luetkens reports a relationship with Bayer HealthCare AG that includes consulting or advisory. Julian A. Luetkens reports a relationship with Philips GmbH that includes speaking and lecture fees. Julian A. Luetkens reports a relationship with Siemens Healthineers AG that includes speaking and lecture fees. Julian A. Luetkens reports a relationship with Norvatis that includes speaking and lecture fees. Julian A. Luetkens reports a relationship with GE Healthcare that includes speaking and lecture fees. If there are other authors, they declare that they have no known competing financial interests or personal relationships that could have appeared to influence the work reported in this paper.

## Data Availability

The datasets used and/or analyzed during the current study are not publicly available, but are available from the corresponding author upon reasonable request.
