# Supplementary material for: Ultrafast deep learning super-resolution single-shot T2-weighted imaging for robust edema visualization in cardiovascular magnetic resonance
Source: J Cardiovasc Magn Reson. 2026 Mar 7;28(1):102708. doi: 10.1016/j.jocmr.2026.102708 (PMC13208670; doi:10.1016/j.jocmr.2026.102708)
Supplement: Supplementary file 1 — Supplementary material [file mmc1.docx]

**SUPPLEMENATRY MATERIAL**


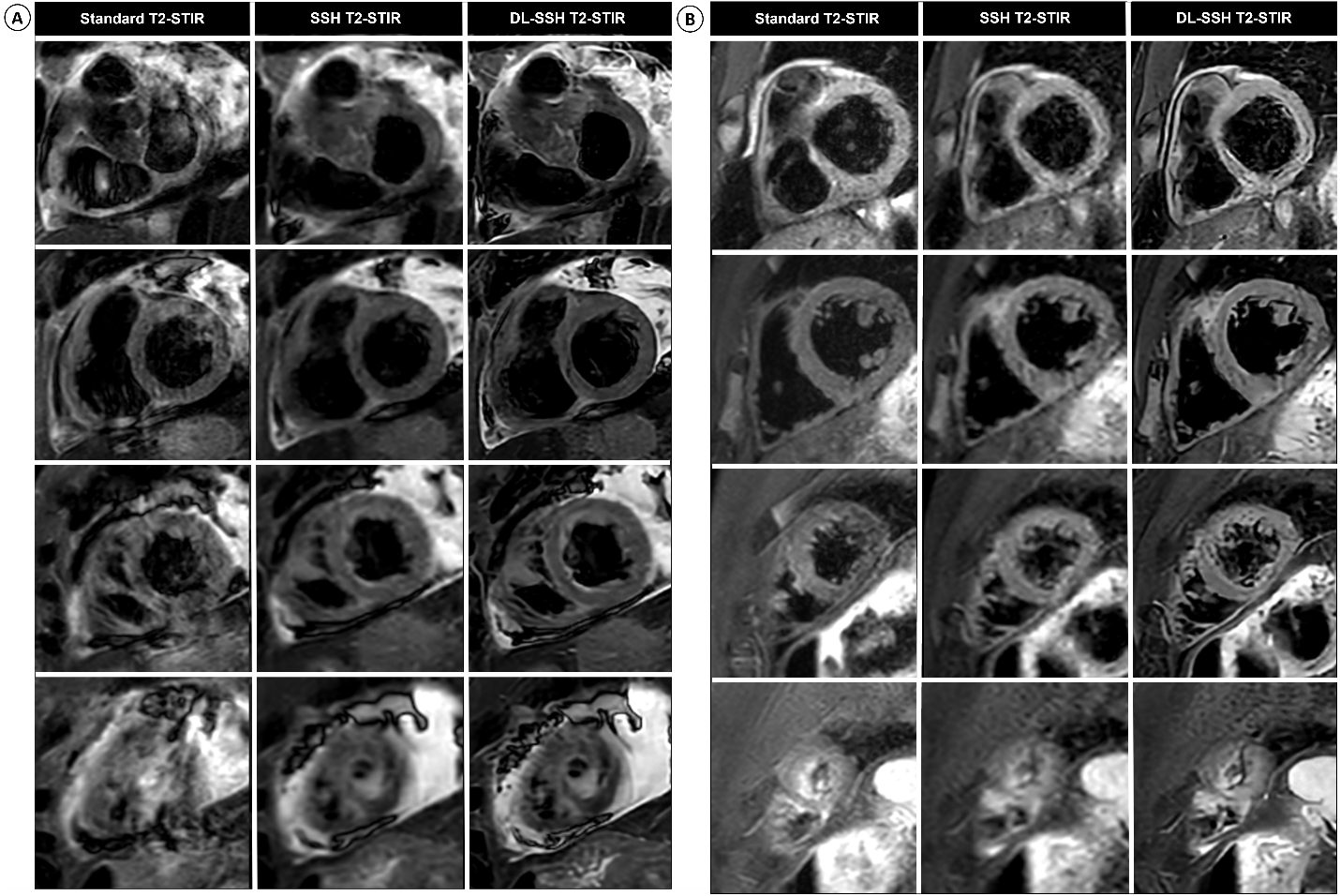


**Figure S1.** Imaging examples of breath-hold segmented T2-STIR (standard), single-shot T2-STIR (SSH T2-STIR), and deep learning–reconstructed single-shot T2-STIR (DL-SSH T2-STIR), across different short-axis levels (basal, mid-ventricular, and apical). (A) 78-year-old participant with pericarditis, large pericardial effusion and insufficient breath-hold capacity. (B) 20-years-old cooperative participant with sufficient breath-holding capacity.
